# Dry Powder Inhalers in the Digitalization Era: Current Status and Future Perspectives

**DOI:** 10.3390/pharmaceutics13091455

**Published:** 2021-09-12

**Authors:** Styliani Xiroudaki, Aurélie Schoubben, Stefano Giovagnoli, Dimitrios M. Rekkas

**Affiliations:** 1 Department of Pharmaceutical Sciences, University of Perugia, 06123 Perugia, Italy; styliani.xiroudaki@studenti.unipg.it (S.X.); aurelie.schoubben@unipg.it (A.S.); 2 Section of Pharmaceutical Technology, Department of Pharmacy, National & Kapodistrian University of Athens, 15784 Athens, Greece

**Keywords:** drug delivery systems, dry powder inhalers, digitalization, smart inhalers

## Abstract

During the last decades, the term “drug delivery systems” (DDSs) has almost fully replaced previously used terms, such as “dosage forms”, in an attempt to emphasize the importance of the drug carrier in ensuring the claimed safety and effectiveness of the product. However, particularly in the case of delivery devices, the term “system”, which by definition implies a profound knowledge of each single part and their interactions, is not always fully justified when using the DDS term. Within this context, dry powder inhalers (DPIs), as systems to deliver drugs via inhalation to the lungs, require a deep understanding of the complex formulation–device–patient interplay. As of now and despite the progress made in particle engineering and devices design, DPIs’ clinical performance is limited by variable patients’ breathing patterns. To circumvent this pitfall, next-generation DPIs should ideally adapt to the different respiratory capacity of individuals across age, health conditions, and other related factors. In this context, the recent wave of digitalization in the health care and industrial sectors may drive DPI technology towards addressing a personalized device–formulation–patient liaison. In this review, evolving technologies are explored and analyzed to outline the progress made as well as the gaps to fill to align novel DPIs technologies with the systems theory approach.

## 1. Introduction

A thorough literature search in Science Direct and PubMed databases evidenced a significant rate increase over the last decade of publications on dry powder inhalers (DPIs), confirming a constant growth of the interest in inhaled medicines. At the same time, several market reports predict a similar growth in the pulmonary administration route, due to an increase in respiratory disease incidence and the need for more effective combination therapies [1,2,3]. The versatility of inhaled therapies, for both local and systemic diseases, justifies the attempt to potentiate the portfolio of innovative pulmonary drug delivery technologies. The United States is leading the global inhalers market, which is expected to reach USD 33,572.9 million by 2023 at a compound annual growth rate (CAGR) of 6.1% during the forecast period [4].

Along with traditional products, smart technologies are emerging to satisfy specific market requirements for cost-effective innovation. In this regard, digital systems have already vehemently penetrated the inhaler market, carrying notorious advantages in the prevention of device use errors. In fact, microprocessors are increasingly implemented in inhalers to assist the patient in proper device handling, with a positive impact on compliance and product satisfaction. However, a real breakthrough in next-generation inhalers may result from the application of smart technologies, allowing device performance to be tailored based on a patient’s variable breathing patterns, while providing user-friendly features and preventing, compensating for, and/or recording potential handling and actuation errors.

Among all the available technologies, the soft mist platform is worth citing due to its patient-centered approach and innovative technology, which is challenging the pressurized metered-dose inhaler (pMDI) and DPI market, thus placing it under the spotlight as a future growth driver [5].

In spite of the winds of change in the inhalation device market, the global inhalers scenario is still lacking innovative technologies for a number of reasons that this review attempts to examine and discuss, with a specific view on DPIs. Moreover, we provide the reader with an updated prospect on the DPI digitalization market as well as shedding light on the application of novel smart technologies and the required steps ahead.

## 2. State of the Art

DPIs are systems capable of delivering a particle-type formulation of an active pharmaceutical ingredient (API) for local or systemic effect via the oral–pulmonary route. They have gained attention as a valid alternative to nebulizers and pMDIs and are currently preferred for the treatment of chronic respiratory diseases [6,7]. DPIs are portable, simple to use, propellant-free delivery systems offering a superior drug physico-chemical stability, deep lung deposition, and the possibility of delivering high drug dosages [8]. With the patient’s inspiratory flow being the primary force for particle deagglomeration and dispersion, they do not require coordination between breathing and device actuation [8].

DPIs are complex systems and their clinical performance depends on fully addressing the harmonic interaction between three important aspects that must be taken into consideration during development: (i) the physico-chemical and engineering properties of the powder formulation; (ii) the design of the device, with a special emphasis on its powder deagglomeration and dispersion capabilities; and (iii) ideally, the strength/pattern of each patient’s inspiratory airflow [9]. 

From a formulation point of view, the powder physico-chemical/engineering properties—namely particle size, particle morphology, hygroscopicity/moisture content, and surface electrostatic charge—are known to determine the particles’ fate and deposition pattern upon inhalation [10]. Such patterns strongly influence the resulting inhaled drug pharmacokinetics (PK) as a consequence of the known dependency of the drug’s fate on the actual deposition site along the respiratory tract. A thorough discussion of such aspects is beyond the scope of this review, and readers can refer to the work of EIKasabgy et al. for more details [11]. In order to reach the lower respiratory tract and optimize pulmonary drug delivery, particle aerodynamic diameter should span between 0.5 μm and 5 μm [12,13]. The preparation of a dry powder for inhalation within this size range is challenging and involves either the mixture of micronized drug with coarse carrier particles, such as lactose, to reduce powder cohesiveness and improve flowability, or particle engineering techniques, including spray-drying, spray-freeze-drying, and supercritical fluid-drying technology [14]. A great deal of work has been carried out in the development of advanced drug delivery systems (DDSs) for DPIs, such as liposomes, nanocomposites, solid lipid nanoparticles, polymeric micro-nano-particles, and microspheres [15]. These advanced delivery platforms seek to overcome the limitations of conventional carrier-based DPIs and a few have already reached the market. Among others, the Pulmosphere^TM^ technology (Novartis, Basel, Switzerland) represents a significant breakthrough in the scenario of engineered inhalable particles. It enables fabrication of low-density, porous particles with a characteristic sponge-like morphology and reduced interparticle cohesion, with improved drug loading (as high as 90% w/w), lung targeting, and dose consistency [16]. Another recent technology, Technosphere^®^ (MannKind Corporation, Westlake Village, CA, USA), consists of a DDS-based on fumaryl diketopiperazine microparticles, which are self-assembled in a mild acidic medium to form microspheres with high porosity and surface area that can be administered without the addition of carrier particles [17]. 

The successful delivery of drugs into the respiratory tract is dependent in part on the integration between device design and formulation. Since the introduction of the first DPI, Spinhaler^®^ (Fisons, Ipswich, UK), devices technology has continued to evolve, and more than 40 different types of devices are currently available on the market [18,19]. They can be broadly classified based on the number of doses they can carry (single-unit dose, multi-dose reservoir, multi-unit dose), the mechanism used for powder aerosolization (passive and active), and the intrinsic resistance characterizing the device (low-, medium-, and high-resistance) [20]. First-generation devices, such as Spinhaler^®^ and Rotahaler^®^ (GlaxoSmithKline, London, UK), are breath-actuated single-unit dose devices, whereas second-generation devices are multi-dose reservoir-type devices, such as Turbohaler^®^ (Astra Zeneca, Cambridge, UK), Easyhaler^®^ (Orion Pharma, Espoo, Finland), Ultrahaler^®^ (Sanofi-Aventis, Paris, France), and MAGhaler^®^ (Boehringer-Ingelheim, Ingelheim, Germany) or multi-unit dose devices, such as Diskhaler^®^ (GlaxoSmithKline, London, UK), Aerohaler^®^ (Boehringer-Ingelheim, Ingelheim, Germany), and Eclipse^®^ (Sanofi-Aventis, Paris, France). Despite their higher cost of production, multi-unit inhalers are likely to ensure better dosage uniformity and a formulation’s chemical stability compared with multiple-dose devices [21]. Recent research has focused on the development of new devices able to ensure dose uniformity and airflow-independent pulmonary delivery [22]. NEXThaler^®^ (Chiesi Farmaceutici, S.p.A., Parma, Italy) is a novel, breath-actuated, multi-unit dose device that provides accurate dose metering and releases the dose only when an inspiratory flow threshold of 35 L/min is reached [23,24]. Newer-generation devices include the so-called ‘’active’’, power-assisted devices, in which external energy forces, such as compressed air, electrical vibration or mechanical impellers, are employed for powder aerosolization, eliminating the need for patient-generated high inspiratory flow rates [25,26]. MicroDose^®^ (MicroDose Therapeutx, Monmouth Junction, NJ, USA) is an active piezoelectric-based DPI that is actuated on sensing a certain inspiratory flow threshold, whereas Aspirair^®^ (Vectura Company, Chippenham, UK) and ResQhaler^TM^ (Aespira Ltd., Moshav Shdema, Israel) employ compressed air as a powder dispersion source [25,27]. The Occoris^®^ (Team Consulting, Cambridge, UK) platform is an active powder aerosolization engine that can be incorporated into various DPIs [27]. Despite being promising and advantageous, its high cost and reduced portability remain a barrier for the widespread use of power-assisted DPIs, and so far none of them has reached the market [28,29,30]. 

## 3. Limitations of Currently Available DPIs

Currently available DPIs vary in aerosolization performance and the fraction of delivered dose in the lungs ranges from approximately 9% to 80% [21]. This high variability and sometimes low lung deposition may be attributed to two major issues: (a) the particles’ strong cohesive forces and inadequate device design that, either taken alone or together, may hamper powder deagglomeration, and (b) the patient’s inability to achieve sufficient inspiratory airflow [21]. Whilst efforts have been focused on advances in both formulation and device design, the patient’s impact on the overall delivery system performance—in terms of inhalation pattern, therapy adherence, and correct inhaler use—is still far from being thoroughly assessed. The currently marketed DPI devices are breath-actuated, and thus a forceful and deep inhalation is needed to disaggregate the powder formulation into particles able to reach the respiratory tract. In general, high patient inspiratory airflows are often associated with improved DPI performance [31]. Nevertheless, because proper dose intake is also dependent on device characteristics, DPI resistance should be accounted for in order to meet the patient’s inspiratory capacity [32]. In fact, selection of DPIs with proper aerodynamic resistance is fundamental for optimal patient–device matching [33], especially for certain patient categories, such as the elderly, children, and individuals with compromised lung function such as asthma, chronic obstructive pulmonary disease (COPD) or cystic fibrosis (CF) disease, who cannot achieve sufficient inspiratory airflows [24]. An out-of-tune patient–device match can often result in incomplete drug emission and/or oropharynx deposition [34,35]. Therefore, it is undeniable that a DPI’s clinical performance depends on the device’s capacity to address a patient’s particular inhalation issue—a feature that cannot however mitigate the high inter- and intra-individual variability in drug delivery [21]. 

Unfortunately, approximately 22–78% of patients with asthma and COPD and 31–53% of CF patients are poorly adherent to their inhaled medications [36,37]. Errors in inhalation techniques occur in a percentage of 6.8–43.2%, and only 10–52% of physicians are adequately informed regarding proper device use [38,39]. The most common clinically significant errors include incorrect positioning of both the device and the head, failure (i) to insert and pierce the capsule (for the capsule-based DPIs), (ii) to exhale away from the device prior to inhalation, (iii) to hold the breath after inhalation, and (iv) to maintain a forceful and deep inspiration. Incorrect inhaler actuation techniques can be associated with patient-related factors, such as age, sex, health condition, and education level [40,41]. A study of a total of 2288 records highlighted that, independent of the type of inhaler used, the strongest association was found between inhaler misuse and older age, lower level of schooling, and inadequate instructions by healthcare providers [42]. As expected, elderly patients with comorbidities, such as Parkinson’s disease and dementia, may be more susceptible to handling errors compared with healthy or younger patients [38].

## 4. Bioequivalence and Bioavailability Issues of DPI Products

Addressing bioavailability/bioequivalence (BA/BE) issues of pharmaceutical products is a critical task for pharma companies. While well established for most oral products—for which the proliferation of the generics market has allowed the exclusivity extension for well-known blockbuster drugs—the issue of BA/BE assessment has only recently been revived as well for orally inhaled and nasal drug products (OINDPs), in light of the patent expirations of a number of landmark inhalation products.

As a consequence, regulatory agencies have focused a great deal of attention on drafting proper guidelines on quality and safety as well as on the BA/BE of inhaled products [43,44,45,46,47,48,49,50], albeit the guidelines are more straightforward for most systemic treatments, as several requirements for BA/BE assessment for locally active pharmaceutical products or specialized delivery systems still remain largely unmet. Overall, OINDPs struggle for a shared consensus on harmonized BA/BE assessment. It was not the aim of this review to provide a detailed survey of all contrasting and divergent approaches and regulations on OINDP BA/BE, as this topic has already been thoroughly debated in recent publications that can be useful to interested readers [51,52,53]. 

An issue worth mentioning though is the different view of the BE problem that persists among the major regulatory agencies. The Food and Drug Administration (FDA) and Japan Ministry of Health recommend comprehensive in vitro–in vivo and clinical characterization to support BE, while the European Medicines Agency (EMA) advises a stepwise BE assessment and accepts in vitro studies provided that there is a total match, in particular in delivered dose uniformity and aerodynamic particle size distribution, between the innovator and the generic [54]. Other agencies, such as Canada Health and the Australian Therapeutic Goods Administration (TGA) line up more with the US, relying mostly on PK and pharmacodynamic clinical studies [53]. Device similarities between generic and innovator products are differently addressed as well, with the FDA being more conservative in recommending a similar number of doses, external operating procedures, size and shape, device resistance, dose indicator/counter, and a patient feedback mechanism. The EU limits recommendations to inhaled volume, handling, and similar resistance.

Such differences raise barriers to BA/BE assessment of inhaled medicines that grow even higher when considering next-generation devices. As will be better discussed later in this review, although new technologies are encouraged by some regulatory agencies, they are not adequately supported by standardization and struggle to find their place in the regulatory framework. This is worsened by the complexity of DPI products, in which device and formulation attributes must converge to provide the target performances. Therefore, both sides should be considered when addressing BA/BE of DPIs. However, guidelines still have a traditional view of DPIs, missing aspects related to technology integration and innovation. Beyond this, the R&D world of inhalable dry powders suffers from years of stagnation in powder engineering, to which the lack of adequate research on new excipients for pulmonary drug delivery has given substantial contribution. To prove it, the current list of accepted materials is limited to lactose, mannitol, glucose, trehalose, phosphatidylcholines, and magnesium stearate [12]. 

While technology is moving fast with the increase in novel, integrated, alternative Good Manufacturing Practice (GMP)-ready approaches that combine next-generation analytical and modeling skills [55], regulations remain behind, gasping to find their way among complex clinical requirements. This trend is exacerbated by an escalation of complexity and costs for OINDP approval and by the scientific gaps that still exist, especially for locally acting DPI products. 

In this regard, beyond recent progress [56], animal models do not support humans [57,58] and sensitive methods for the assessment of clinical efficacy as well as in vitro/in vivo correlation for inhaled medicines are still lacking [59]. In this scenario, the clinical endpoint approach is strongly biased by the not always clear correlation between efficacy and selected clinical endpoints. This risk is higher for therapies in which the drug deposition pattern is directly linked to efficacy. Konstan et al. and Ramsey et al. [60,61] have clearly pointed out this problem in CF therapy, where, as with other inhaled medicines such as bronchoprotectants, the forced expiratory volume (FEV1) is usually employed as a clinical efficacy endpoint. In these studies, FEV1 did not change significantly when treating patients with liquid aerosols or dry powders, despite the known different lung deposition behavior of the two forms. Darquenne et al. [62] ascribed such non-correlation to the features of the spirometry method, it being responsive only to proximal deposition effects. This evidence, in line with EMA guidelines [49], warns of a potential insensitivity of efficacy studies when BE is assessed on a response scale. As anticipated, this issue is more relevant when a localized effect is sought, such as for inhaled antimicrobial therapies, for which current guidelines show conflicting recommendations [63,64,65]. In such cases, treatment complexity, the lack of standardized protocols, and the appropriateness of comparator products make selection of proper clinical endpoints even more challenging. Moreover, surrogate analyses, such as sputum concentration, which is used to measure clinical drug concentrations in the lungs, is highly variable and likely unreliable [66]. 

Confirming the above, Montgomery et al. [67] questioned the use of FEV1, exacerbation or sputum analysis, and patient-reported outcomes as primary or surrogate endpoints in inhaled antibiotic therapies due to their lack of correlation with clinical efficacy. In this case, additional confirmatory endpoints were required to avoid clinical outcome and side effect confounding.

Khoubnasabjafari et al. [68] recently postulated that the use of the exhaled breath condensate test, which reflects alveolar lining fluid composition, as an alternative to current testing methods for BE assessment. This method may help address some of the above issues; nevertheless, a robust validation is required in order to confirm its clinical merit among different inhalation products.

Confounding is a real problem in clinical trials for DPIs in particular, where the risk of errors in inhaler handling by the patient can be high. Discerning patient’s handling errors from actual clinical effects is not easy, especially in phase III trials where assistance and support to patients cannot be granted. Here is where digital systems can really make an impact by allowing monitoring and control over all phases of clinical studies [69]. How digitalization may help BA/BE assessment of DPI products is insightfully discussed in the following sections.

## 5. Digitalization: From Nebulizers and pMDIs to DPIs 

In light of all the above considerations, two interrelated critical questions arise: Is there space for novelty in inhaled medications? If yes, which would/should be the direction? In this regard, digitalization is a fast growing and promising technology that could support patient-centered care, while offering realistic solutions not only for patients, but also for healthcare providers and pharma companies. Digital technology can improve clinical outcomes by enhancing adherence and minimizing a patient’s errors through cell-phone applications connected with the device, which provide useful tools such as reminders and step-by-step guidance for the proper usage technique. From the other side, physicians can track in real time a patient’s compliance to the therapeutic regimen or symptom worsening and consequently prevent severe disease exacerbations and improve the patient’s quality of life [70]. This is of crucial importance for chronic respiratory diseases since it is often challenging to distinguish patients with refractory disease from those with poorly controlled disease due to low adherence or improper inhaler use [71]. This real-time information is also of paramount importance for the health technology assessment (HTA) organizations and regulatory agencies in validating the safety and effectiveness of drug products under “real life” conditions. Digitalization can improve monitoring of the current pharmacovigilance system and economically benefit pharma companies through increased patient’s adherence [70]. 

As mentioned above, for drug–device combinations, and particularly for inhalation products, errors related to patient device usage and delivery variations, as well as defective data collection, may provide distorted information concerning therapeutic efficacy in clinical trials [69]. Hence, the introduction of digital tools in clinical trials can contribute to a better understanding with regard to drug–device combination efficacy, ensuring that the collected data are not influenced by poor adherence and administration issues [69]. These digital tools could include devices and audio-based technologies to monitor medication adherence and connected wearables to track the activity of patients and evaluate the effect on clinical efficacy, as well as companion apps to capture patient-reported outcomes [72].

“Smart” inhalers, as termed in the related literature, which provide reliable and objective feedback mechanisms regarding medication adherence and inhalation technique, have been added to the existing therapeutic arsenal for the treatment of chronic respiratory diseases—namely, asthma, COPD, and CF. They can be divided into two categories: add-on devices, in which the e-module is externally attached to the inhaler, and originally integrated devices, in which the e-module is incorporated inside the device [73]. Their development started in the late 20th century from simple devices able to track inhaler usage (Table 1). The Nebulizer Chronolog (NC) (Forefront Engineering Corporation, Denver, USA) was the first adherence-monitoring device of inhaled medication to receive FDA approval in 1982 [74]. It was designed as an enhanced case surrounding the inhaler canister, and it was able to record not only inhaler actuations but also the exact date and time of every actuation, storing roughly 4000 events [74]. In the following years, the market was invaded by other “smart” inhalers, both nebulizers and pMDIs. At first, “intelligent” nebulizer systems, such as the I-neb^®^ Adaptive Aerosol Delivery (AAD^®^) device (Philips Respironics, Murrysville, PA, USA), AKITA^®^ (Activaero GmbH, Gemunden, Germany), and AeroEclipse^®^ (Monoghan/Trudell Medical International, London, ON, Canada) were launched. They ensured optimal drug delivery by detecting a patient’s breathing pattern and releasing the aerosol only during the inspiratory phase, thus preventing losses during exhalation, for which conventional nebulizers are notorious [75,76,77,78].

Doser^TM^ (Meditrack Inc., Hudson, MA, USA), Smart Mist^®^ (Aradigm Corporation, Hayward, CA, USA), MDILog^TM^ (Westmed Technologies Inc., Englewood, USA), Smart Inhaler Tracker (Adherium Ltd., Auckland, New Zealand), and Smart Track^TM^ (Adherium Ltd., Auckland, New Zealand) were some of the first digitalized pMDIs [36,107]. Except for the Doser, all of them provide records regarding date and time of actuation, as well as specific reminders through ringtones [107]. Furthermore, MDILog and SmartMist can additionally detect inhalation, and the latter provides immediate feedback on the inhalation technique: a red light is displayed when inhalation is too rapid, a green light when inhalation is adequate, and no light when inhalation is too weak [107]. Subsequently, the Propeller^®^ device (ResMed, San Diego, CA, USA) introduced further innovation, embedding Global Positioning System (GPS) functionality, which enabled detection of the exact location where patients experienced asthma exacerbation episodes and thus mapped high-risk areas and potential environmental triggering factors [107]. Propeller^®^ add-on sensors are compatible with a wide range of pMDIs, such as Flovent^®^ (GlaxoSmithKline, Brentford, UK), Dulera^®^ (Merck, Kenilworth, NJ, USA), QVAR^®^ (Teva Pharmaceutical Industries, Petah Tikva, Israel), and Ventolin^®^ HFA (GlaxoSmithKline, Brentford, UK) [108]. In 2015, the Propeller Platform (PP) received FDA clearance for the only SMI marketed: the Respimat^®^ (Boehringer Ingelheim, Ingelheim am Rhein, Germany) [109]. In 2020, the FDA approved a customized Propeller sensor for Symbicort^®^ (AstraZeneca, Cambridge, UK), for which the pharmaceutical company had previously marketed an FDA-approved device, the SmartTouch^TM^ (currently called as Hailie^®^ sensor) for Symbicort^®^ in collaboration with Adherium Ltd. (Auckland, New Zealand). FindAir ONE (FindAir, Kraków, Poland), a “smart” add-on device for pMDI inhalers, was launched in the European market in 2019 and provides, among other benefits, useful information regarding the pollen level, air quality, and weather conditions, to help asthma patients predict an asthma attack-promoting environment [90,91]. Further advances were achieved by the Intelligent Control Inhaler (ICI) (3M Drug Delivery Systems, Saint Paul, MN, USA), which originally was an integrated breath-actuated pMDI, capable of registering the dose only upon the patient’s correct inhalation rather than on device actuation and also capable of monitoring the inhalation flow rate, aiming to minimize technique errors [110,111].

## 6. Marketed and under Development Digitalized DPIs

As discussed hereafter, the research and development of “smart” pMDIs has paved the way to the development of digital DPIs (Table 2).

Although first conceived as pMDI sensors, the PP has recently received FDA clearance as add-on sensors for DPIs as well. Propeller^®^ sensors dedicated to Diskus^®^ (GlaxoSmithKline, Brentford, UK), Ellipta^®^ (GlaxoSmithKline, Brentford, UK), and Neohaler^®^ (Novartis, Basel, Switzerland) received FDA approval in 2015, 2016, and 2018, respectively [36,109,119]. In 2020, the European Commission (EC) announced Enerzair^®^ Breezhaler^®^ (Novartis, Basel, Switzerland) approval for all EU member states, as well as the UK, Iceland, Norway, and Liechtenstein [115]. It is indicated for the maintenance treatment of asthma in adult patients not adequately controlled with a combination of a long-acting beta2-agonist (LABA) and a high dose of an inhaled corticosteroid who experienced at least one asthma exacerbation event in the last year [130]. The optional digital companion consists of a customized electronic Propeller^®^ sensor that can be attached to the base of the inhaler and that records actuations as well as the whirring noise of the spinning capsule during inhalation [130]. The sensor can be linked with the Propeller^®^ app via Bluetooth [131]. In addition, the app sends the patient specific reminders for the inhaler’s use and monitors adherence over time [132].

In 2018, Adherium Ltd. launched the Hailie^®^ solution (previously known as Smartinhaler™) in the US, after its FDA 510(k) over-the-counter clearance to enable sales direct to consumers [116]. It involves an add-on sensor, developed for pMDIs as well (Table 1), that can be attached to the inhaler and records data regarding medication usage. The sensor can be paired with the Hailie^®^ app on a mobile device, where patients can set up specific reminders and track medication usage [92]. In June 2021, Adherium submitted an FDA 510(k) application for a next-generation Hailie^®^ sensor able to capture physiological data as well [133]. Hailie^®^ add-on sensors dedicated to Diskus^®^ (SmartDisk™) and Handihaler^®^ (SmartHandy™) were recently launched [117].

On the European side, Respiro was approved by the EMA and received the European Conformity (CE) mark in 2018. The Respiro digital health platform consists of a sensor able to monitor device usage and to help patients to adopt correct actuation maneuvers. Respiro was marketed the same year for three different products: Spiromax^®^, Nexthaler^®^, and Ellipta^®^ [120].

Another add-on device that has been evaluated in different studies and trials [71,125,126,127] is the Inhaler Compliance Assessment (INCA). It is a CE-marked acoustic recording device manufactured by Vitalograph Ltd. INCA registers the time and technique of inhalation by acquiring the audio of the inhalation act to identify wrong procedures, such as exhalation or too short an inhalation time. Audio files are collected and analyzed to understand patient adherence and inhalation technique suitability, with the objective of improving treatment and establishing patient training requirements.

The Diskus^®^ technology officially entered the digital world in 2020, when Aptar Pharma acquired the Cohero Health portfolio [134]. Cohero Health has conceived different digital tools and technologies such as the HeroTracker Sensor class B digital device. This add-on sensor was combined with the Diskus^®^ DPI as a reminder to ensure patient adherence to a therapeutic regimen. The sensor also records and monitors actuations once it is connected via Bluetooth to the BreatheSmart app [135].

In the early 2000s, a digital version of a well-known product, the Advair Diskus DPI, was tested by adding Diskus Adherence Logger add-on sensor technology [121], which was characterized by small dimensions, low cost, and extended battery duration (6 months). The Adherence Logger was found able to record the date and time of use to guarantee patient adherence to treatment. Nevertheless, to the best of our knowledge, no further development of this product has been reported, suggesting that this technology has been discontinued for some reason.

Another add-on device is the Verihaler, also developed for pMDIs (Table 1). It incorporates a small microphone that records inhalation acoustics from DPIs. Based on a proprietary algorithm, information such as peak inspiratory flow rate (PIFR) and dose emission can be extracted from the acoustic file. It can be linked via Bluetooth to a medical-grade app allowing the collection of evidence connecting a potential clinical condition with issues of device misuse and therefore to poor or inadequate adherence [104,106]. Despite its promise, Verihaler has not yet reached the market.

Teva Pharmaceutical Industries Ltd. (Petah Tikva, Israel) obtained FDA approval in 2018 for the first all-in-one digital DPI with built-in sensors and Bluetooth technology [112]. The ProAir^®^ Digihaler^®^ is a digital rescue inhaler approved for use in four-year-old and older patients to treat or prevent bronchospasm with reversible obstructive airway disease, such as asthma and COPD, and for the prevention of exercise-induced bronchospasm [136]. It incorporates a built-in electronic module—namely, a pressure sensor, a wireless transmitter, and a processor [137]. The pressure sensor consists of barometric type Micro-Electro-Mechanical Systems (MEMS) able to detect pressure changes smaller than 1 Pa [137]. The pressure sensor port is attached to the device mouthpiece and can detect various inhaler activities, including the mouthpiece cover opening [137]. ProAir^®^ Digihaler^®^ has a dose counter attached to the actuator, which at the beginning of the treatment displays the number 200, which corresponds to the total available actuations. When the dose counter reaches 20, the color of the numbers changes to red, whereas when the dose counter reaches 0, the whole background color changes to solid red [138]. In addition, if no information regarding the dosage is sensed within a pre-scheduled dosing period time, a specific reminder can be sent to the patient and/or the caregiver [137]. The device can be connected with a smartphone application using a Quick Response (QR) code located on the top of the inhaler. When connected to the inhaler, the app allows the patient to monitor all MEMS-recorded inhaler information, including PIFR and inhalation volume, daily inhaler events, and checks for updates on weather and environmental conditions [139]. In this way, patients are able to review and share data with their healthcare provider, which may contribute to a more informed discussion regarding disease management [140]. A pilot study on 360 patients highlighted that a predictive model based on data recorded by the device, which included inhaler use, PIFR, volume inhaled, time to peak flow, and inhalation duration, was effective in predicting an imminent asthma exacerbation [141]. Overall, the average number of albuterol daily inhalations over a period of five days before an asthma exacerbation event resulted in being the main predictive factor [141]. The Digihaler^®^ “family” was expanded with the approval by the FDA of two digital products: the AirDuo^®^ Digihaler^®^ and the ArmonAir^®^ Digihaler^®^ in 2019 and 2020, respectively. They are indicated for the treatment of asthma in patients aged twelve years or older. The AirDuo^®^ Digihaler^®^ can be used for symptom control, such as wheezing, whereas the ArmonAir^®^ Digihaler^®^ is indicated for longer-term treatment [113,114]. They both incorporate the same technology as the ProAir^®^ Digihaler^®^.

In 2020, Respiro was integrated into the well-known RS01 single-dose DPI to produce the digital RS01X DPI [70]. It tracks medication usage via its built-in sensor and can be connected to Amiko’s Respiro app, which additionally provides patients with personalized guidance on proper use techniques and medication reminders [70]. Furthermore, the RS01X automatically detects and records patient-generated inhalation parameters, such as inhalation flow rate and duration [70]. 

To the best of our knowledge, the Digihaler^®^ family and RS01X complete the existing portfolio of marketed integrated DPIs. However, other digital DPIs with built-in technology and noteworthy features have been also developed. 

More specifically, the USSC-03 device is manufactured as an electronic system able to monitor time, date, and inhalation profile to evaluate treatment adherence. It is provided with a sensor able to measure the pressure drop inside the device so that the blister is not opened until a pressure drop of about 1.5 kPa is detected. This allows wrong powder deposition following a poor inspiratory act to be avoided [73,129]. 

Another product with distinctive features is the Inspiromatic™ DPI. Noteworthy advancement results from its active DPI features, being equipped with a powder fluidization system activated at low inhalation flow rate (7 L/min) and a data logger to record patient’s performance. It was evaluated in a comparative study against Aerolizer^®^ as a reference product in asthmatic children [128]. This active DPI was well accepted, safe, and showed a performance comparable with the Aerolizer^®^, but with a superior potential in handling pediatric patients as well as patients with low inspiratory capacity.

The Diskhaler^®^ and Breezhaler^®^ are devices with a long market tradition that are under development to go digital. In particular, the first paper reporting the use of an electronic Diskhaler dates back to 1997, when it was used to study treatment compliance in asthmatic adults [123]. This smart device registers both blister perforation and airflow, which are indicators of dose administration [124]. The electronic version of the Breezhaler^®^ [122] is slightly larger but shares the same performance, with the additional capacity of monitoring and recording inhalation use.

## 7. Regulatory Standards in DPI Digitalization 

DPIs are considered drug–device combination products by the EMA, which recently issued a draft guideline on the quality requirements for drug–device combinations [142,143]. Based on this, it is important to distinguish an integral device from a non-integral one. The first has to meet the following three characteristics: (i) the device and the medicinal product form a single integral product, (ii) it is intended for use in the given combination, and (iii) it is not reusable [144]. Therefore, medical devices that are co-packaged with a medicinal product are non-integral devices. Consequently, DPIs already loaded with drug doses in blister (multi-unit dose) or those with a powder reservoir (multi-dose reservoir) are considered integral devices. Conversely, DPIs used to administer a single-dose unit are classified as non-integral devices. Since the action of the medicinal compound(s) is of principal importance in DPIs, they have to comply with Directive 2001/83 CE or Regulation (EC) No 726/2004 on medicinal products [145]. For marketing authorization of an integral device, it is necessary to follow Article 117 of Regulation (EC) No 745/2017. In particular, the dossier should include a Declaration of Conformity (DoC) or a certificate for the medical device from a EU notified body. If this documentation is not provided together with the marketing authorization dossier, it is mandatory to include a DoC to Annex I of the medical device regulation reporting the general safety and performance requirements for medical devices [146]. For non-integral medical devices, the CE mark is mandatory, in accordance with the medical device legislation. Then, due to technological advancements and emerging technologies, DPIs should also have a measuring or metering function. To guarantee treatment efficacy, it is of paramount importance that an innovation, among the wide range of inhalers with different designs, does not leave the patient confused and disoriented [38,147]. For this purpose, the role of the EMA is to evaluate the “quality, safety, and efficacy” of the medicinal product along with device safety and performance. Regarding any software combined with the digital inhaler, at the European level, little is reported in the literature. In particular, the EU Court of Justice issued a judgment at the end of 2017 attesting that a software can be classified as a medical device under EU law if certain conditions are met [148]. If the software is specifically developed to be used for diagnosis, prevention, monitoring, treatment, or alleviation of disease according to the Directive 93/42/EEC, the software will fall under the medical device Directive [148,149]. The need for a judgment on this matter reveals the complexity of the regulations for medical devices and their interpretation. It is also important to remember that Regulation (EC) No. 745/2017 was fully effective as of 26 May 2021. On the regulatory standards of software combined/contained in medical devices, the FDA is more explicit with regard to specific guidance for the content of premarket submissions for software, which was published approximately 15 years ago [150]. Moreover, mobile medical applications are also regulated by the FDA, as reported in the last issue of the policy for device software functions and mobile medical applications [151,152]. According to the FDA, DPIs fall within the definition of combination products [153,154]. In particular, drug-specific inhalers can reach the market after approval of a new drug application (NDA), while a solitary device can follow two different routes: (i) NDA or (ii) the 510(k) with the FDA Center for Devices and Radiological Health [155] in cases in which the drug is already approved by the Center for Drug Evaluation and Research [156]. Several FDA departments are involved in the approval of combination products, and this may create difficulties for an applicant. The complex approval process is a common global issue for digital DPIs. As reported by Sven Dethlefs from TEVA, the approval process is long and, while fitness devices are not FDA-regulated products, the software development for DPIs is required to consider many similar aspects (e.g., documenting action, notification,…), and it is necessary to interact with several departments [157]. It would therefore be desirable to have a clear EU/US guideline that establishes harmonized procedures for the approval for these innovative digital inhalers.

In Japan, medical devices are regulated by the Act on Securing Quality, Efficacy and Safety of Pharmaceuticals, Medical Devices, Regenerative and Cellular Therapy Products, Gene Therapy Products and Cosmetics, effective since November 2014 [158]. To date, no specific document is available on the approval process of digitalized products, and they are likely to be evaluated individually during review process.

Since 31 January 2020, the UK was formally out of the EU, but the European pharmaceutical law continued to apply to the UK until the end of 2020. Brexit had a strong impact on the EMA, which worked hard since March 2019 to relocate physically to Amsterdam and to avoid medicine shortages as much as possible. The EMA also issued guidance for companies involved in Brexit. Inhalers or medicinal product–medical device combinations produced in the UK would need to be tested in European facilities in order to be released in Europe as imported products, since the UK is now considered to be a separate country. All of these aspects have to be followed carefully, with substantial reference to the official website of the EU and UK governments [159,160].

Another aspect, not closely related to the regulation of digitalized products but of paramount importance, deals with the protection of sensitive information of patients who have to be monitored [152]. In this regard, the World Health Organization (WHO) has published several papers based on its eHealth global survey [161,162].

## 8. Gaps to Be Filled to Reach Digitalization

Terms such as “smart” DDSs and the like, while technologically feasible today, should be used with caution, since they reflect a product’s clinical performance in the real world, once approved and commercialized.

We believe that now is time to define what “smart” really means when referring to pharmaceutical products and devices at the health system level, and thus, regulatory guidelines should be put forward for discussion and expedited adoption of such a concept.

**Figure 1:**
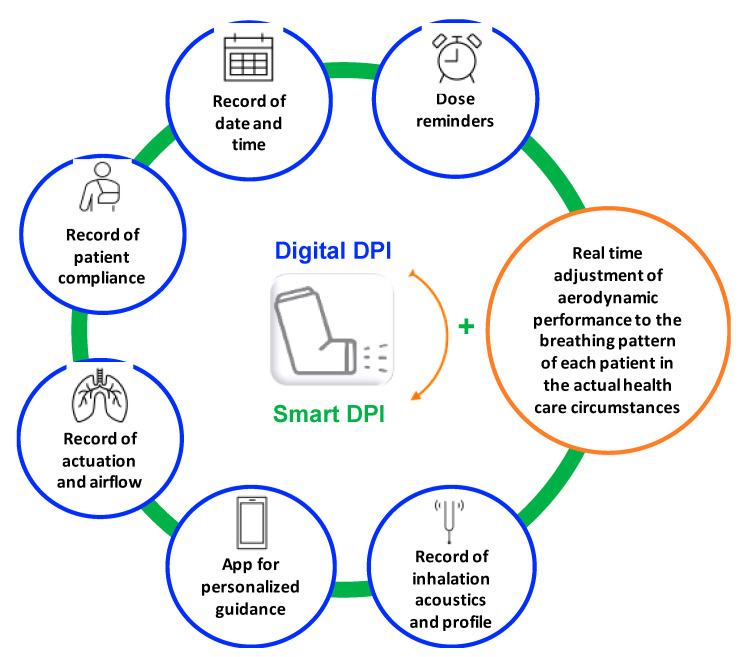
Summary of the available digital functions of the current digitalized DPIs arsenal. The requirement for “smart” device features, according to a generally recognized definition, also defines the gap that exists today between digital and smart devices.

There is a well-recognized gap between what is recorded in clinical trials before marketing authorization of drug products compared with post-approval evidence, which negatively affects the safety/effectiveness index of drug products. This “lost in translation” scientific drawback has to be bridged with advanced tools such as artificial intelligence (AI) and Big Data, as defined in the Industry 4.0 wave of change. Inhalation devices could be a starting point for delivering real advancements in filling the gap between efficacy and effectiveness for patients’ benefit. In this regard, substantial contribution could stem from the growing arsenal of DPI digital functions that, as summarized above and in Figure 1, are evolving to erase the distance between simple digitalized and actual smart systems.

Within this context, we propose a series of assertions to be considered by health community experts and patient advocacy groups as a “starting seed” to raise awareness and discussion:

- The “smart” definition and its proper use in pharmaceutical products. This term and its equivalents should be used only when meeting predefined conditions set by the regulatory authorities. Silverio-Fernández et al. [163] contributed to the definition of “smart device”. Based on a selected literature search, they initially identified five key features corresponding to the term “smart device”: autonomy, connectivity, context-awareness, user-interaction, mobility, and data storage. The first three core aspects appeared in their review of the related literature twice as much when compared with the last two. A “smart” device should be able to interact with patients, with other stakeholders of the health system, and of course with other devices. Moreover, it should be able to produce reliable data, and their patterns should be available in real time to all stakeholders in the health system. Above all, however, the “smart device” must, when taken as a whole and respecting systems theory, provide a pragmatic solution to a real problem, i.e., meeting a real unmet need of the patient, an unmet need that is predefined by the health authorities. Applying the above concept to inhalation DDSs, a device could be authorized as “smart” only when its ability to predict and adjust to the different breathing patterns of patients is scientifically proved and approved by regulators.
- The Internet of Things (IoT) aspect of “smart” coupled with AI and machine learning capabilities will eventually be a change driver in transforming the currently passive and deferred pharmacovigilance system to an active one, permitting real-time evaluation and interventions for assuring continuous monitoring of the safety/effectiveness index of drug products across their lifecycle.
- The health system will be able to pay suppliers based on real-world valid data, relating the pricing/reimbursement schemes with the pragmatic performance of DDSs.
- This will also change the landscape of clinical trials, as they will be on-going through a product’s lifecycle and data will be shared by the patient with all concerned parties under real-time conditions.
- Reviewing the “European 2020 Pharmaceutical Strategy” and its updates, it is now officially recognized that there are “*unmet needs due to the absence of commercial interest and limitations of science*” and that “*research priorities should be aligned to the needs of patients and health systems*”. To overcome this gap quickly and efficiently, many aspects need to be changed both at the tactical level and strategy-wise. In spite of the current rush toward personalized medicine, most inhaled products are developed in a non-patient-centered fashion without accounting for actual individual needs. Therefore, greater effort should be focused on the understanding of patients’ unmet needs rather than the researchers’ citation portfolio. This is the transformation that must be “technologically” driven when designing next-generation inhalation devices. A “smart” device should facilitate real solutions for a given patient need, one that is not “invented” in order to “justify” a novelty claim. The implemented “smart” technology is an input in the whole process, which when interacting with other factors, such as selection of API, excipients, and device design, enhances the DPI’s capacity to address a real unmet need.
- Given the thousands of publications related to inhalation delivery, perhaps it is time to reconsider how many of them have actually contributed to practical advancements by impacting patient lives. The advantage of the scientific community being “data super-rich” is at the same time its problem because its abundance hampers the transformation of this big basket of a myriad of mixed data into pharmaceutical products and services that are valuable to patients.

Last but not least, the establishment of the smart concept will also allow the definition of quality in pharmaceuticals to be corrected. In fact, in regulatory guidelines the terms “quality”, “efficacy”, and “safety” are proposed as separate terms; thus, their obvious interconnection is missing, while it is scientifically absolutely solid that quality equals safety and effectiveness.

The technological/digital evolution of DDSs has to be translated into inhalation devices in a way that substantially improves their clinical performance in comparison with the current state of the art. To achieve this, in our opinion the following are fundamental steps in order to identify the gaps to be filled with “next-generation” devices:

- Redefine the scope and stay tuned with the patient’s unmet needs. The one who is responsible for imparting this new message is the scientist, who through deep knowledge and high ethics has to carefully listen to patient’s needs so as to quickly address them.
- These needs have to be communicated, discussed, and adopted by the scientific community. There are a lot of meetings, many of them financially supported by the EU, which could easily serve this purpose.
- A clear “position paper” has to be constructed to effectively communicate these unmet needs of patients to health system stakeholders in real time.
- This “consensus paper” should be an official “compendium”, like the pharmacopoeias, which every stakeholder consults and has to comply with before designing a new inhalation product or an extension of an approved one.
- The regulatory authorities have to adopt this as a “gold standard”, to differentiate the submissions needing attention and priority from those that are the mere *n*th copy product of the original.
- HTA committees should make this “standard” visible to all stakeholders and make clear that the health system will only pay for the real, scientifically solid, delivery of value to the patient and not for a “copy and paste” approach. This is a major paradigm shift for paying for the value delivered to the patient and not paying based on sales volume discounts.
- Redefine, at the regulatory level, the definition of quality. It is not rational to adopt in a regulatory document a “chemistry-oriented” definition, which never existed in the related literature on quality, but instead to promote the quality-by-design initiative, in which quality is presented correctly, as was proposed by Juran three decades ago. Moreover, as our understanding of the gaps is progressing, the term “efficacy”, reflecting what is achieved in clinical trials, has to be replaced with the term “effectiveness”, which underlines what is realized after a product’s approval, under real-world conditions. This means adopting the correct approach, which is that quality equals safety and effectiveness, with the note that quality is continuously monitored, in real time, over the whole product lifecycle.
- The regulatory list of what is called “quality guidelines” has to be revisited and the first guideline, to start the journey towards fulfilment of the patient’s unmet needs, should be the “Pharmaceutical Quality System” known as International Council of Harmonization Q10 (ICHQ10). This guideline should also be revised based on the following two recommendations. The first should be a precaution to readers “not to proceed with the study of the rest of quality guidelines if ICH Q10 (now as ICHQ1) is not fully understood”. The second is to bring on a brief, but well-presented introduction to “systems theory”, which must be implemented as a prelude to the pharmaceutical quality system.
- In health systems there is at least one paradox. The two ends of the pharmaceutical supply chain are represented by the producer on the one side and the patient on the other side, supported by regulatory agencies and HTA committees. Being actual payers for the delivered therapeutic value, patients should be pivotal leaders. If so, health authorities should set a new array of standards as defined above. Particularly, payers should be in the position to entrust manufacturers to supply an inhalation device which, among the other known regulatory requirements, is capable of maintaining optimal delivery performance regardless of a patient’s breathing patterns. This is a standard practice adopted in other fields, where governments pay manufacturing companies for the development of specific technological solutions. The above mentioned scheme could also be applied to publicly funded research programs in the health sector.

## 9. Perspectives after Digitalization

W.E. Deming coined a very important laconic phrase: “No aim? No System!” It is now time for the so called “patient-centered approaches” to be translated into realistic health solutions. The above aim being clear, the strategy of the pharmaceutical industry and researchers should assure the satisfaction of predefined unmet medical needs. 

The payer should take the lead in setting the therapeutic targets to be met and success should be measured against publicly pre-announced requirements that reflect the scientific gaps to be filled for patient’s benefit.

Within this context the digitalization era in the health system should be framed and communicated to all concerned parties, and this has to be carried out by the regulatory bodies on behalf of the patients.

Regulators should shift from an “auditing–monitoring” passive attitude to an attitude of being active health system players, re-writing and imposing the rules of the reimbursement schemes upon entirely new bases:

- Promoting and demanding real innovation with fast and reliable approval procedures for drug products that meet preset therapeutic targets;
- Assuring real-time safety and effectiveness of drug products in the pragmatic world of everyday clinical practice.

The COVID-19 pandemic revealed specific weaknesses in the above context, and the intellectual power of academia along with the pharmaceutical industry should be aligned and focused on the diseases/clinical cases for which knowledge is insufficient or lacking. 

Recognizing and communicating what we do not know is the first and sincere step forward.

This review, although it specifically tries to address the gaps in DPIs digitalization, comports with the authors’ intention to promote a broader debate as well so as to encourage a cultural change in the current approaches to the research and development of pharmaceutical products.

## Figures and Tables

**Table 1:** Marketed and under-development digitalized nebulizers, pMDIs, and SMIs in alphabetical order (not intended to be fully exhaustive).

Brand Name	Device	Add-On	Integral	Company Name	Approval/ Launched/Publication Year	References
ADHERO	pMDI	✓		Lupin Limited/Aptar	2019 †	[79]
AeroEclipse^®^	nebulizer		✓	Monoghan-Trudell Medical International	2006 *	[80]
AKITA^®^	nebulizer		✓	Activaero GmbH	2009 *^,^**	[81]
Breelib^TM^	nebulizer		✓	Vectura/Bayer	2016 **	[82,83]
CapMedic^®^	pMDI	✓		Cognita Labs	2020*	[84,85]
CareTRx^®^	pMDI	✓		Teva Pharmaceutical Industries Ltd.	2014 †	[86,87]
Doser^TM^	pMDI	✓		Meditrack Inc.	1994 *#	[74]
eMDI^TM^	pMDI		✓	H&T Presspart—Cohero Health	2016 †	[88,89]
FindAir ONE	pMDI	✓		FindAir	2019 **	[90,91]
Hailie^®^	pMDI	✓		Adherium Ltd.	2018 *	[92,93,94]
HeroTracker^®^	pMDI	✓		Aptar Pharma (Cohero Health)	2015†	[95]
I-neb^®^ Adaptive Aerosol Delivery (AAD^®^)	nebulizer		✓	Philips-Respironics	2006 *^,^**	[37,75,96]
Inspair	pMDI	✓		Biocorp	2016 †	[97]
Intelligent Control Inhaler (ICI)	pMDI		✓	3M Drug Delivery Systems	2016	[98,99]
MDILog^TM^	pMDI	✓		Westmed Technologies Inc.	1997 *#	[74]
Nebulizer Chronolog	pMDI	✓		Forefront Engineering Corporation	1982 *#	[74]
Pneumahaler	SMI		✓	Pneuma Respiratory	2017	[100,101]
Propeller^®^	pMDI	✓		ResMed (Propeller Health)	2014 *^,^**	[102]
Smart Inhaler Tracker	pMDI	✓		Adherium Ltd.	2006 #	[74]
Smart Mist^®^	pMDI	✓		Aradigm Corporation	1996 *#	[74]
SmartTrack^TM^	pMDI	✓		Adherium Ltd.	2009 *	[103]
T-Haler^®^	pMDI		✓	Cambridge Consultants	2012	[104,105]
Verihaler	pMDI	✓		Sagentia Innovation	2010	[104,106]

* FDA, ** EMA approved, † marketed, # currently withdrawn from the market/use limited to the academic environment.

**Table 2:** Marketed and under-development digitalized DPIs in alphabetical order (first rows are dedicated to marketed products; not intended to be fully exhaustive).

Brand Name	Add-On	Integral	Characteristics	Company Name	Approval/Launched/ Publication Year	References
Digihaler^®^ for			Pressure sensor (peak inspiratory flow, inhaled volume). Records date and time of use. Sends dose reminders.			
ProAir^®^		✓		Teva Pharmaceutical Industries Ltd.	2018 *	[112]
AirDuo^®^		✓		Teva Pharmaceutical Industries Ltd.	2019 *	[113]
ArmonAir^®^		✓		Teva Pharmaceutical Industries Ltd.	2020 *	[114]
Enerzair^®^ Breezhaler^®^	✓		Records date and time of use. Records inhalation acoustic. Sends dose reminders.	Novartis- ResMed (Propeller Health)	2020 **	[115]
Hailie^®^ sensor for			Records date and time of actuation. Provides audiovisual reminders.	Adherium Ltd.	2018 *	[116]
Diskus^®^ (SmartDisk™)	✓			GlaxoSmithKline	2019 *	[117]
Handihaler^®^ (SmartHandy™)	✓			Boehringer Ingelheim	2019 *	[117]
HeroTracker^®^ Sensor for	✓		Records actuations. Sends dose reminders.	Aptar Pharma (Cohero Health)	2020 (2015)	
Diskus				GlaxoSmithKline	2019	[118]
Propeller^®^ sensor for			Records date and time of actuation. Innovative GPS functionality enables to identify triggers of exacerbation.	ResMed (Propeller Health)		[74]
Diskus^®^	✓			GlaxoSmithKline	2015 *	[109]
Ellipta^®^	✓			GlaxoSmithKline	2016 *	[119]
Neohaler^®^	✓			Novartis	2018 *	[36]
Respiro for			Records device use. Provides personalized guidance.	Amiko SRL		[120]
Spiromax^®^	✓			Teva Pharmaceutical Industries Ltd.	2018 **	
Nexthaler^®^	✓			Chiesi Farmaceutici S.p.A.	2018 **	
Ellipta^®^	✓			GlaxoSmithKline	2018 **	
RS01X		✓	Records device use. App provides personalized guidance.	Berry Global Healthcare -Amiko SRL	2020 **	[70]
Diskus Adherence Logger	✓		Records date and time of use.	-	2004	[121]
Electronic Breezhaler		✓	Records device use.	Novartis	2018	[122]
Electronic Diskhaler		✓	Records blister perforation and airflow.	-	1997	[123,124]
Inhaler Compliance Assessment (INCA)	✓		Records inhalation audio	Vitalograph Ltd.	2016 **	[71,125,126,127]
Inspiromatic™		✓	Records patient compliance.	OPKO Health Inc.- (Inspiro Medical Ltd.)	2014	[128]
USSC-03		✓	Records date and time of use. Records inhalation profile.	Novartis	2016	[73,129]
Verihaler	✓		Records date and time of use. Records inhalation acoustic.	Sagentia Innovation	2010	[104,106]

* FDA, ** EMA approved.

## Data Availability

Not applicable.
